# RNA-Seq and Single-Cell RNA-Seq Analyses of Tilapia Head Kidney in Response to *Streptococcus agalactiae* and *Aeromonas hydrophila*

**DOI:** 10.3390/ani15202951

**Published:** 2025-10-11

**Authors:** Qi Li, Zulin Fang, Zhengshuang Li, Xinxian Wei, Youchuan Wei

**Affiliations:** 1 Key Laboratory of Aquatic Healthy Breeding and Nutrition Regulation of Guangxi Universities, College of Animal Science and Technology, Guangxi University, Nanning 530004, China; 2 Key Laboratory of Aquaculture Genetic and Breeding and Healthy Aquaculture of Guangxi Academy of Fishery Sciences, Guangxi University, Nanning 530021, China

**Keywords:** Nile tilapia, *Streptococcus agalactiae*, *Aeromonas hydrophila*, RNA-Seq, scRNA-Seq

## Abstract

**Simple Summary:**

High-throughput sequencing has broad applications and holds great promise for elucidating immune mechanisms in lower vertebrates. In this study, we conducted, for the first time, a comparative analysis of the changes in tilapia lymphocytes following infection with different bacterial pathogens and discovered differential differentiation of monocytes/macrophages (Mos/Mφs) in response to Gram-positive and Gram-negative bacterial infections. We hope that these data will contribute to elucidating the antibacterial response mechanisms and the evolution process in bony fish.

**Abstract:**

High-throughput sequencing has significantly advanced the exploration of fish immune mechanisms, enabling a more detailed understanding of immune responses and their underlying molecular pathways. In this study, we applied comparative transcriptomics and single-cell RNA sequencing to investigate the immune mechanisms of tilapia in response to different pathogenic bacteria. Our results demonstrated that nonspecific cytotoxic cells (NCCs) and monocytes/macrophages (Mos/Mφs) mounted the most pronounced responses to both *Streptococcus agalactiae* and *Aeromonas hydrophila* infections. Moreover, Mos/Mφs exhibited distinct differentiation patterns depending on the bacterial challenge. Collectively, these findings offer new insights into the antibacterial immune strategies of lower vertebrates.

## 1. Introduction

*Streptococcus agalactiae* and *Aeromonas hydrophila* are among the most common Gram-positive and Gram-negative bacterial pathogens in tilapia and typically cause septicemia, meningitis, enteritis, and skin ulceration, leading to significant economic losses in the tilapia farming industry [1,2,3,4]. For example, the tilapia exports in Malaysia faced the risks of losing up to approximately 40% of their export value without the test-kit intervention targeted at *Streptococcus* disease [5]. Moreover, *S. agalactiae* is often referred to as group B streptococci (GBS) in clinical medicine and primarily infects newborns and pregnant women, leading to septicemia and bacteriuria [6].

High-throughput sequencing has greatly advanced our understanding of gene expression and immune response patterns in tilapia during bacterial infections over the past two decades [7,8,9,10,11,12]. The bulk RNA-Seq is currently the most mature and widely applied sequencing method, since its strength lies in the highly efficient and cost-effective profiling of global gene expression in tissues. Currently, an urgent need exists for higher-resolution analyses to reveal the heterogeneity of tilapia responses to different pathogenic bacteria [13]. Thus, the single-cell RNA-Seq (scRNA-Seq) is rapidly emerging due to the advantage in precisely resolving cellular heterogeneity down to the single-cell level and is expected to act as an in-depth discovery tool for revealing novel cell types and the precise cellular mechanisms of immune responses.

Therefore, in this study, we conducted both bulk transcriptome and single-cell transcriptome analyses of tilapia head kidney lymphocytes (HKLs), given the crucial immunological role of the head kidney in fish and building on extensive prior research. Previous studies have demonstrated that the tilapia head kidney functions as an innate immune center during bacterial infection [14,15,16], that HKLs can be resolved at the single-cell level [17,18,19], and that numerous antiviral molecules are present within HKLs [20]. In addition, in vitro stimulation models using tilapia HKLs and lipopolysaccharide (LPS) or lipoteichoic acid (LTA) revealed a powerful antibacterial response, such as the rapid release of inflammatory factors and activation of the immune pathways within a few hours post-infection [14,17].

Consequently, the RNA-Seq and scRNA-Seq analyses indicated that nonspecific cytotoxic cells (NCCs) and monocytes/macrophages (Mos/Mφs) mounted predominant responses to both Gram-positive and Gram-negative bacteria. Moreover, Mos/Mφs displayed distinct trajectories in response to the two bacterial infections, whereas NCC differentiation did not show such divergence. Together, this study provides fundamental data for investigating the behavior and mechanisms of fish lymphocytes in response to pathogenic bacteria.

## 2. Materials and Methods

### 2.1. Fish and Bacteria

Nile tilapia (*Oreochromis niloticus*, 100 ± 10 g) was obtained from a commercial farm in Nanning City, China. Fish were acclimated in recirculating aquaculture systems with proper aeration, and the water temperature was maintained at 28 °C. After two weeks of acclimation, healthy fish were selected for experimentation following the criteria, including steady swimming, good appetite, even respiration, unwounded body and fins, bright eyes, well-proportioned abdomen, and undetectable with *Streptococcus agalactiae* and *Aeromonas hydrophila* through the spread-plate technique.

Preserved strains of *S. agalactiae* and *A. hydrophila* were reactivated by overnight incubation in brain–heart infusion (BHI) broth and Luria–Bertani (LB) broth, respectively, at 28 °C [8]. Bacterial cultures were harvested by centrifugation, washed, and resuspended in sterile phosphate-buffered saline (PBS).

### 2.2. Ethics Approval Statement

All animal experiments were approved by and conducted in accordance with the guidelines of the Ethics Committee of Guangxi University (GXU-2025-058).

### 2.3. Challenge and Statistics of Survival Rate

A total of 40 tilapia were randomly divided into three groups: the control group (*n* = 10; intraperitoneally injected with 100 μL of sterile PBS), the *S. agalactiae*-infected group (*n* = 15; intraperitoneally injected with 100 μL of *S. agalactiae* [5 × 10^7^ CFU/mL]), and the *A. hydrophila*-infected group (*n* = 15; intraperitoneally injected with 100 μL of *A. hydrophila* [5 × 10^7^ CFU/mL]). The survival rate (SR) was calculated daily for 5 days using a previously described iterative formula [21]:

$$\mathrm{SR}=\left(1-\frac{Dead\;fish}{Survival\;fish_{\left(previous\;day\right)}-\;sampled\;fish}\right)\times {\mathrm{SR}}_{\left(previous\;day\right)}\times 100\%$$

### 2.4. Head Kidney Lymphocyte Samples Collection

Three fish from each group were anesthetized and sacrificed at 24 h post-infection (hpi). Head kidney tissues were dissected, minced, and then passed through a 40 μm cell strainer (Beyotime, Shanghai, China) before being placed in L-15 medium. Cell suspensions were layered onto a 50% Percoll gradient (Solarbio, Beijing, China) and centrifuged with a swing rotor at 800× *g* for 10 min at 4 °C. Cells located at the surface of the Percoll layer were gently aspirated, collected by centrifugation, washed, and resuspended in L-15 medium.

Subsequently, the cell density was adjusted to 10^7^ cells/mL, and 1 mL of the cell suspension was collected by centrifugation and used for RNA-Seq. For scRNA-Seq, three HKL samples were prepared, each consisting of a pooled mixture of three individual samples (1 mL each).

### 2.5. RNA-Seq and Bioinformatics Analysis

Total RNA was extracted using RNAiso Plus (TaKaRa, Dalian, China). mRNA was enriched, fragmented, and reverse-transcribed into cDNA. The resulting cDNA was end-repaired, ligated to Illumina sequencing adapters, amplified by PCR, and sequenced on the Illumina NovaSeq 6000 platform (Gene Denovo Biotechnology Co., Guangzhou, China). The subsequent RNA-seq data processing and bioinformatics analyses were performed through the online tools (https://www.omicsmart.com/, https://www.omicshare.com/tools/, accessed on 20 May 2025) operated by the Gene Denovo Biotechnology Co., following our previous studies [8,17]. Raw reads (sequencing depth = 6 G) were filtered to obtain high-quality clean reads using fastp (v0.18.0). Paired-end reads were mapped to the *O. niloticus* reference genome (Ensembl release 113) using HISAT2 (v2.4.0).

Gene expression levels were quantified and normalized as fragments per kilobase of transcript per million mapped reads (FPKM). Differentially expressed genes (DEGs) were identified using DESeq2 (v1.26.0) with the criteria |log_2_(fold change)| ≥ 1, *p* < 0.05, and false discovery rate (FDR, Q value) ≤ 0.05. KEGG pathway enrichment analysis was performed using the KEGG Automatic Annotation Server (KAAS).

### 2.6. ScRNA-Seq Data Processing

The cell density was adjusted to 1 × 10^6^ cells/mL for single-cell sequencing using the 10 × Genomics platform (Gene Denovo Biotechnology Co., Guangzhou, China). The subsequent scRNA-seq data processing and bioinformatics analyses were performed through the online tool (https://www.omicsmart.com/) operated by the Gene Denovo Biotechnology Co., following previous studies [17,18,19].

Briefly, the Raw single-cell 3′ library data (sequencing depth = 120 G) were demultiplexed into FASTQ files using the “cellranger mkfastq” (https://www.10xgenomics.com/support/cn/software/cell-ranger/latest/analysis/inputs/cr-mkfastq, accessed on 20 May 2025) tool from Cell Ranger (v3.1.0), resulting in the Illumina sequencer’s base call files (BCLs) for each flow cell directory into FASTQ files. Reads were aligned to the *O. niloticus* reference genome (Ensembl release 113) using Spliced Transcripts Alignment to a Reference (STAR), which compares the cDNA fragments (Read 2) used in the double-end sequencing of Illumina to the reference genome. The type of reads (exons, introns, or intergenic regions) was identified based on the corresponding GTF annotation. A read is considered aligned to the transcriptome and annotated as a transcriptome-aligned read if it aligns to an exon of a transcript and both share the same direction. Among the transcriptome-aligned reads, those that map to only one gene are defined as uniquely mapped. Only unimapped reads were used for unique molecular identifiers (UMIs) counting. Low-quality barcodes and UMIs were removed using “cellranger count”. Cell barcodes were required to exactly match the barcode sequences in the reference database, allowing for at most one mismatch, which could only occur at low-quality base positions. The software then performed error correction, and any barcodes not meeting this criterion were filtered out. The criteria for UMI filtering: (1) not a homopolymer; (2) without N; and (3) not containing bases with base quality < 10.

Processed data were further analyzed using the Seurat R package (v3.1.1). Cells were filtered based on the following criteria: (1) the number of genes per cell was less than 200 or greater than 4000; (2) the number of UMIs per cell was >15,000; and (3) the percentage of mitochondrial (Mito) genes was over 10%. Gene expression data were log-normalized for global scaling with the following formula: expression level (gene A) = log (1 + (UMI A ÷ UMI Total) × 10,000). Batch effects were corrected with Harmony. Principal component analysis (PCA) was performed in Seurat to refine the expression matrix. Cell-to-cell distances were calculated based on significant principal components, and a shared nearest neighbor graph was constructed. Edge weights were refined using Jaccard distances. Cells were clustered using the Louvain algorithm to maximize modularity, and results were visualized with uniform manifold approximation and projection (UMAP).

### 2.7. Cell Annotation and DEGs Identification

Lymphocytes were reclustered after erythrocyte removal, which was based on hemoglobin gene expression (ENSONIG00000032930 and ENSONIG00000039402, expression values < 0.1). The resulting 20 initial clusters were classified into six lymphocyte populations based on the expression of marker genes. Differential expression analysis between the cell subpopulations was performed using the FindMarkers function in Seurat with the likelihood-ratio test (threshold value = |log_2_(fold change)| ≥ 0.36 and *p* < 0.01) [19], and KEGG annotations were performed as described above.

### 2.8. Upregulated Gene Identification

Based on the distribution of DEG numbers across different cell subpopulations, the NCC and Mos/Mφ subsets were found to harbor a strikingly higher number of DEGs, exceeding 4000 and 2000, respectively. Consequently, these two specific cell subpopulations were prioritized for subsequent in-depth analysis. The Mann–Whitney U test conducted by the FindMarkers function in Seurat was used to identify upregulated genes in NCC and Mos/Mφ subclusters using thresholds of |log_2_(fold change)| ≥ 0.36 and *p* < 0.01, requiring detection in >25% of cells within the target subcluster [19].

### 2.9. Cell Trajectory Analysis

Single-cell trajectories of NCCs and Mos/Mφs were reconstructed using Monocle 2 with the cell and gene expression matrices as input [18]. Trajectories were plotted separately for NCCs and Mos/Mφs, with cells ordered using the DDRTree method and the root state reassigned. The analysis utilized the highly variable genes identified through the standard Seurat workflow as the ordering gene set. Cells were subsequently ordered along the reconstructed trajectory using the orderCells function, generating pseudotime values that represent their progression states. The DDRTree method effectively modeled the complex branching differentiation process, providing both the trajectory structure and continuous pseudotime estimates for downstream analysis. Differential gene expression analysis along the pseudotime axis was performed using the differentialGeneTest function in Monocle2. DEGs were identified within each state using thresholds of *p* < 1 × 10^−5^ and Q < 1 × 10^−5^. KEGG enrichment analysis of selected DEGs was performed as described above. Genes with significant expression changes before and after branch points along the trajectories were also identified and analyzed.

## 3. Results

### 3.1. RNA-Seq of HKLs Reveals Different Responses to Two Bacterial Infections

**Figure 1:**
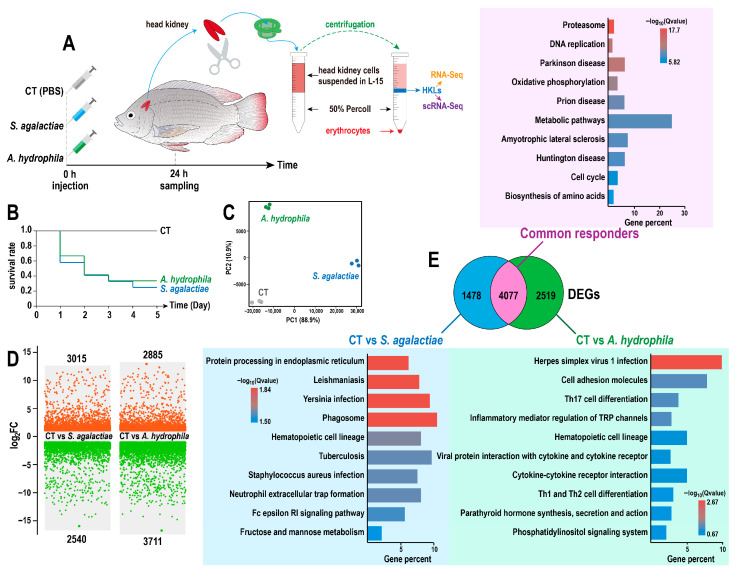
Transcriptome analysis of tilapia head kidney lymphocytes (HKLs) during bacterial infection: (**A**) Workflow of bacterial challenge and sample collection. Tilapia were intraperitoneally injected with sterile PBS, *S. agalactiae*, or *A. hydrophila*, and the head kidney lymphocytes (HKLs) of tilapia (*n* = 3 per group) were sampled at 24 h post-infection (hpi) for RNA-Seq and scRNA-Seq. (**B**) Survival curves following infection. (**C**) Principal component analysis (PCA) of RNA-Seq expression profiles. (**D**) Number of differentially expressed genes (DEGs). (**E**) Top 10 enriched KEGG pathways, including common DEGs and bacteria-specific DEGs.

A challenge experiment was conducted to examine the mRNA expression profiles of head kidney lymphocytes in tilapia, and two well-studied pathogenic bacteria, *S. agalactiae* and *A. hydrophila*, were applied (Figure 1A). Bacterial infection caused substantial mortality, with survival rates below 40% within five days (Figure 1B). Subsequently, nine transcriptome libraries generated a total of 404.4 megabases (Mb) of clean reads, with an average Q30 score of 96.8% and an average mapping ratio of 91.5% to the tilapia genome. The PCA results indicated that the mRNA expression profiles of HKLs at 24 hpi presented obvious differences, coinciding with peak mortality (Figure 1C). Next, the DEGs between the control group and the two infected groups were determined, resulting in 5555 DEGs against *S. agalactiae*, while 6596 DEGs responded to *A. hydrophila* (Figure 1D). Among these, 4077 DEGs were common to both infections, while 1478 and 2519 DEGs were specific to *S. agalactiae* and *A. hydrophila*, respectively (Figure 1E). KEGG enrichment analysis of these DEGs highlighted pathways such as proteasome, protein processing in the endoplasmic reticulum, and herpes simplex virus 1 infection (Figure 1E).

### 3.2. ScRNA-Seq of HKLs Indicated NCCs and Mos/Mφs Were Central Defenders

**Figure 2:**
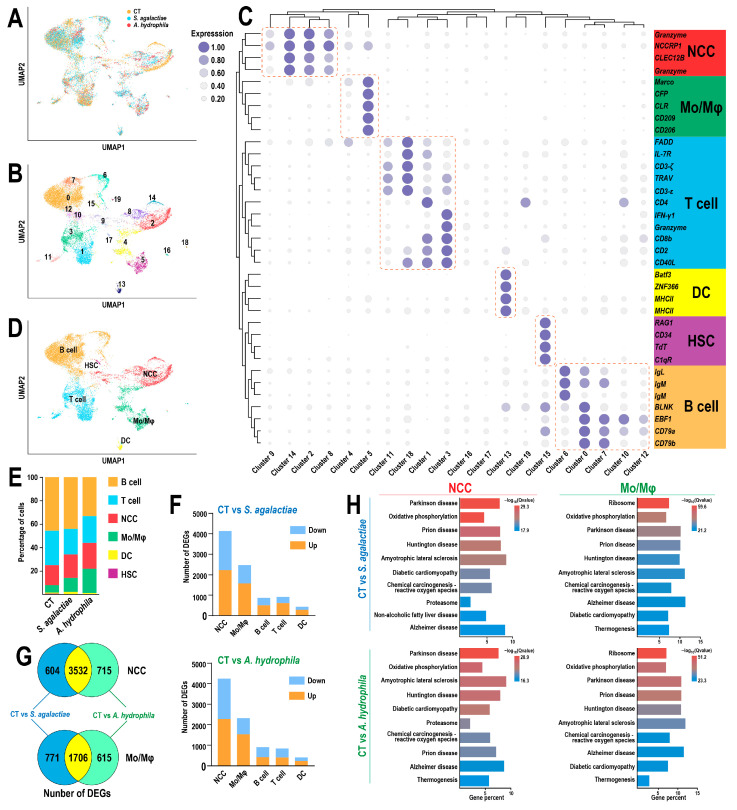
Single-cell transcriptome profiling of tilapia HKLs: (**A**,**B**) Distribution of three head kidney lymphocytes and the original 20 clusters visualized by uniform manifold approximation and projection (UMAP). (**C**) Heatmap showing the clustering of recognized markers. (**D**) UMAP representation of six identified leukocyte types. (**E**) Proportion of each leukocyte population. (**F**) Number of DEGs per population. (**G**) DEGs identified in NCCs and Mos/Mφs. (**H**) Top 10 enriched pathways of DEGs in NCCs and Mos/Mφs.

Three single-cell transcriptome libraries were generated, identifying six distinct subpopulations, including hematopoietic stem cells (HSCs), B cells, T cells, nonspecific cytotoxic cells (NCCs), monocytes/macrophages (Mos/Mφs), and dendritic cells (DCs), based on the expression of established marker genes (Figure 2A–D and Figure S1). Specifically, a total of 20,191 immune cells (CT group: 6731 cells, *S. agalactiae* group: 8133 cells, *A. hydrophila* group: 5327 cells) were captured, sequenced, and identified. Consequently, 3715 NCCs, 2958 Mos/Mφs, 4722 T cells, 7517 B cells, 238 DCs, and 169 HSCs were screened. Notably, the proportions of NCCs and Mos/Mφs increased following infection (Figure 2E), and these two cell types exhibited the highest number of DEGs (Figure 2F). Consistent with RNA-Seq results, a substantial proportion of common DEGs in response to *S. agalactiae* and *A. hydrophila* were observed in NCCs (72.8%) and Mos/Mφs (55.2%) (Figure 2G). However, the top 10 significantly enriched pathways of DEGs in NCCs and Mos/Mφs were highly similar regardless of whether the infection was caused by a Gram-positive or Gram-negative bacterium (Figure 2H), such as Parkinson’s disease and Oxidative phosphorylation pathways in NCCs and Ribosome and Oxidative phosphorylation pathways in Mos/Mφs.

### 3.3. Characterization of HKLs Biomarkers Against Bacterial Infections

**Figure 3:**
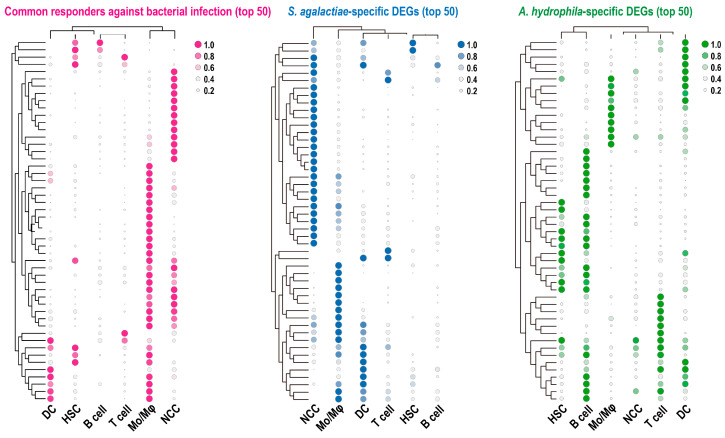
Heatmap of the top 50 DEGs across different leukocyte types, including common responders and *S. agalactiae*- or *A. hydrophila*-specific DEGs, which are identified in RNA-Seq analyses.

The distribution of DEGs from RNA-Seq was further assessed, and the heatmaps illustrated the clustering of typical common responders, as well as *S. agalactiae*-specific and *A. hydrophila*-specific DEGs (Figure 3). The top 50 DEGs responsive to bacterial infections, along with the *S. agalactiae*-specific DEGs, were predominantly enriched in NCCs and Mos/Mφs. In contrast, the *A. hydrophila*-specific DEGs were broadly detected across cell types, except for NCCs.

### 3.4. Differentiation of NCCs Against Bacterial Infections

**Figure 4:**
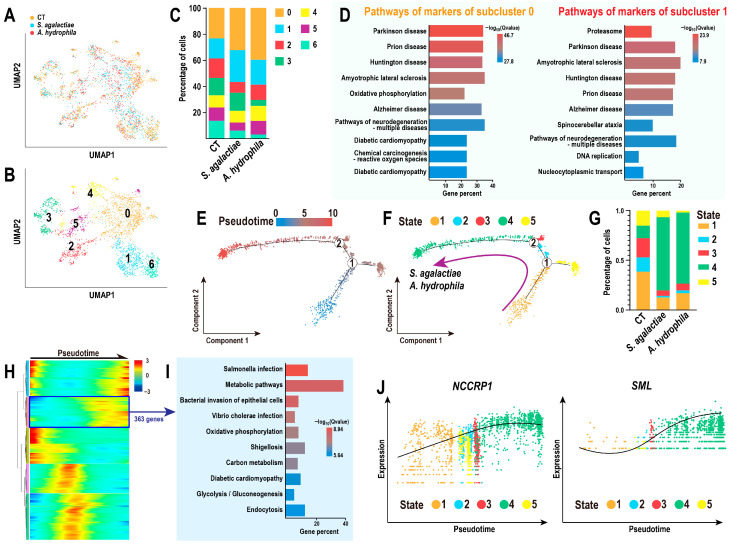
NCC differentiation trajectories during bacterial infection: (**A**) UMAP distribution of NCCs. (**B**,**C**) Identification and proportion of subclusters of NCCs. (**D**) Top 10 enriched pathways of markers (upregulated genes) of subclusters 0 & 1. (**E**) Pseudotime trajectory of NCCs with two branch nodes. (**F**,**G**) Distribution and proportion of five NCC differentiation states. (**H**,**I**) Top 10 enriched pathways of DEGs correlated with NCC differentiation under bacterial infection. (**J**) Expression of key NCC biomarkers, NCCRP1 and SML.

A subsequent investigation was conducted to characterize the behavior of NCCs under infection, given their intense response. Seven subclusters of NCCs were identified (Figure 4A,B), and their relative proportions were assessed (Figure 4C). Meanwhile, KEGG annotation of the upregulated markers in subclusters 0 and 1 was performed, as these subclusters expanded significantly following infection. This analysis indicated that the enriched pathways were primarily associated with Parkinson’s disease, the proteasome, and prion disease (Figure 4D). Subsequently, the pseudotime trajectory of NCCs was defined (Figure 4E), and the states of the cellular states were quantified and visualized through scatter plots and histograms (Figure 4F,G). This analysis revealed that the NCCs at stage 4 were the predominant population post-infection, regardless of whether the infection was caused by *S. agalactiae* or *A. hydrophila*, as denoted by the purple arrow lines in Figure 4G. Consequently, a unified differentiation trajectory of NCCs in response to both bacterial infections was established (Figure 4F). Moreover, 2134 DEGs along the differentiation trajectory of NCCs were identified, clustered, and visualized through a heatmap (Figure 4H, Table S1), including a cluster of 363 genes that were highly correlated with NCC responses to bacterial infection. KEGG annotation indicated that these DEGs were primarily enriched in pathways related to bacterial infection and metabolic processes (Figure 4I). Additionally, several canonical biomarkers and effectors of NCCs were identified, such as NCCRP1 and SML (Figure 4J).

### 3.5. Disparate Trajectories of Mos/Mφs Against S. agalactiae and A. hydrophila

**Figure 5:**
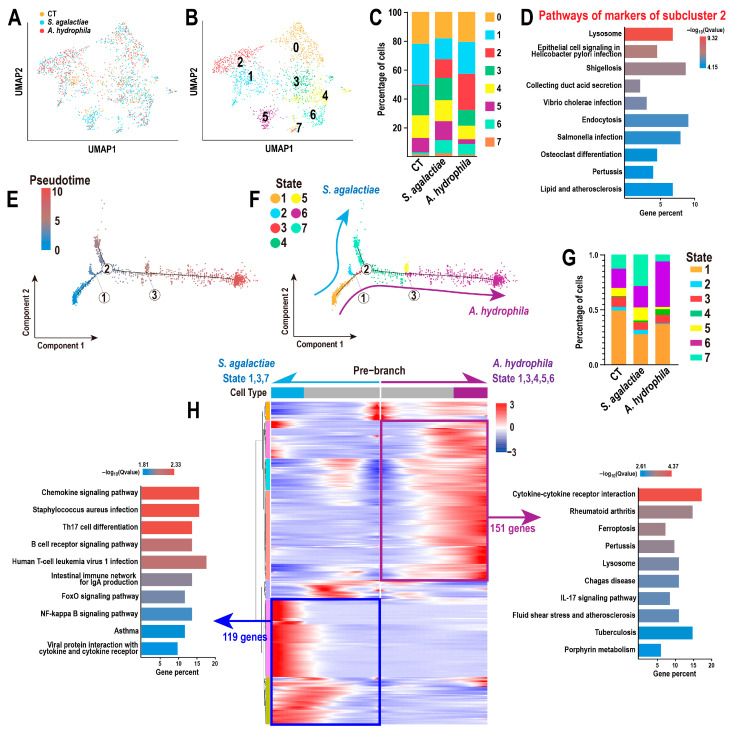
Mos/Mφ differentiation trajectories during bacterial infection: (**A**) UMAP distribution of Mos/Mφs. (**B**,**C**) Identification and proportion of Mos/Mφ subclusters. (**D**) Top 10 enriched KEGG pathways of markers in subcluster 2. (**E**) Pseudotime trajectory of Mos/Mφs with three branch nodes. (**F**,**G**) Distribution and proportion of seven Mos/Mφ differentiation states. (**H**) Top 10 enriched pathways of branch-dependent DEGs at branch node 2.

Similarly, the composition situation of Mos/Mφs and the variation of subcluster proportion were analyzed, resulting in the identification of eight subclusters (Figure 5A,B). Notably, Mos/Mφs in subcluster 2, characterized by 944 upregulated markers, showed marked expansion following bacterial infection (Figure 5C). KEGG annotation indicated that these markers were primarily involved in lysozyme-related processes and the antibacterial responses of endothelial cells (Figure 5D). The pseudotime trajectory of Mos/Mφs was constructed, and three branching nodes were identified (Figure 5E). Mos/Mφs at states 7 and 6 were highly associated with protection against *S. agalactiae* and *A. hydrophila*, respectively (Figure 5F,G), as indicated by the blue and purple arrow lines in Figure 5F, and their distinct trajectories diverged from branch node 2 (Figure 5F). Subsequently, 306 branching differential genes were identified at branch node 2, including 119 and 151 genes that may play key roles in Mos/Mφs responses to *S. agalactiae* and *A. hydrophila*, respectively (Figure 5H, Table S2). Moreover, KEGG annotation analysis of these branch-dependent genes suggested that chemokines and cytokines were the principal biological processes mediating Mos/Mφ differentiation in response to Gram-positive and Gram-negative bacteria, respectively.

## 4. Discussion

Our current study provides new insights into the behavior and mechanisms of fish lymphocytes in response to *S. agalactiae* and *A. hydrophila* infections. To achieve this goal, both conventional RNA-Seq and advanced scRNA-Seq approaches were employed to assess the heterogeneity of HKLs under bacterial infection. Compared with the previous sampling strategy that used the entire head kidney for transcriptome analysis [8], the parallelism and reproducibility of the HKL gene expression matrix across individuals were improved, thereby reducing errors in subsequent analyses. This was also recorded in numerous in vitro models using tilapia HKLs [14,17,21,22,23]. Consequently, the PCA results and DEG analysis highlighted the substantial heterogeneity of tilapia HKLs under different bacterial infections, while a considerable proportion of common DEGs was also observed. Notably, KEGG annotation of the common responders revealed fundamental defense pathways against bacterial infection in fish, including ubiquitination-mediated protein degradation [12], DNA replication [24], and oxidative phosphorylation [25]. In addition, several *S. agalactiae*-specific and *A. hydrophila*-specific pathways were identified, such as endoplasmic reticulum-related processes associated with *S. agalactiae*, which were noticed during the analysis of the combined effects of high-fat diet feeding and *S. agalactiae* infection, and might further regulate the inflammatory response [26]. Besides, the interferon and Toll-like receptor pathways are associated with *A. hydrophila* and induce proinflammatory effects and the apoptosis process [27,28].

Meanwhile, the atlas of tilapia HKLs against bacterial infection was evaluated, and this analysis exhibits good stability and consistency of lymphocytes proportion, compared to the previous study [17], through the minor adjusted cell isolation methods. In particular, the 34% Percoll gradient was removed, and the centrifugal acceleration and duration were optimized to 800× *g* and 10 min, respectively, resulting in the simplification of the preparation process of the cell suspension. Besides, the tissue located at the surface of the 34% Percoll layer was previously regarded as cell fragments [17,19], which was negated by the trypan blue staining result. In addition, compared to the previous study, a slightly increased proportion of T/B cells was determined in this study (65% vs. 54%), in accord with the small size and lower density features of fish T/B cells [29,30]. Subsequently, most DEGs of lymphocytes were identified in NCCs and Mos/Mφs, while the dominant DEGs of HKLs that respond to PolyIC stimulation were detected in NCCs and T cells [20]. To date, there are some studies that have characterized the biomarkers or effectors of NCCs and Mos/Mφs in tilapia, mainly based on the classical RNA-Seq approach using whole head kidney tissue and in vitro models with the isolated immune cells. For example, the regulation of complement C3 [31] and Siglec7 [22] on inflammatory response and phagocytosis of tilapia Mos/Mφs, and the mediation of transferrin on the killing activity of NCCs [32]. However, the real-time dynamic responses of Mos/Mφs and NCCs inside the fish body remain insufficient, which might provide more details of the behavior and differentiation of Mos/Mφs and NCCs triggered by the cell-to-cell interactions. Our present study indicated the universal roles of NCCs in tilapia innate immunity, whereas the Mos/Mφs and T cells present preferences. Although few differences in the KEGG annotation analysis result of DEGs across NCCs and Mos/Mφs that underwent bacterial infection, the noticeable discrepancy in the number of DEGs of these two cell subpopulations emphasized the significant heterogeneity present.

Furthermore, the distribution patterns of representative DEGs identified through RNA-Seq were analyzed, reinforcing the pivotal defensive roles of NCCs and Mos/Mφs during bacterial infection. Notably, adaptive immune cells exhibited clear activation at 24 hpi in response to *A. hydrophila*, as evidenced by the upregulation of known B-cell markers (IgM, CD22, EBF1) and T-cell effectors (perforin-1), consistent with the KEGG annotation findings. A similar situation was observed in vaccinated tilapia, where the violent activation of the T/B cells postimmunization, especially for the vaccine against *A. hydrophila* [33]. Also, a strong T/B-cell response in teleost fish induced by *A. hydrophila* is a widely observed and studied phenomenon. For instance, bacterial infection can strongly induce the activation of CD3γδ T cells [34] and promote B-cell differentiation [35] in grass carp. In summary, our results suggest that RNA-Seq remains a classical and reliable method that continues to provide valuable insights.

Subsequently, the developmental trajectories of NCCs under bacterial infection were fitted, leading to the identification of hundreds of differentiation-related antibacterial genes, including the universal NCC marker NCCRP1. However, the precise immunological role of NCCRP1 and even the definitive existence of NCCs remain subjects of debate. For example, a study on common carp suggested that NCCRP1 likely functions as a ubiquitin-like protein in cytotoxic cells [36], similar to findings in mammals [37]. Moreover, recent studies in tilapia and large yellow croaker revealed that NCCRP1 is a cytoplasmic protein that is strongly activated following pathogenic infection [7,38]. In this study, although some ubiquitination-related processes were identified in NCCs during bacterial infection, as well as the studies in zebrafish and rainbow trout indicated the function of ubiquitination against bacterial infection, such as the regulation on NF-κB pathway [39] and the inhibition of inflammation [40], more extensive and robust ubiquitination-mediated degradation events were observed during the antiviral responses of NCCs [17,19,20]. In addition, NCCs in fish were identified in the 1980s [41] and were subsequently regarded as the precursor or homolog of mammalian natural killer cells (NK cells), which share many common features, such as the fundamental cytotoxicity usually conducted by the perforins and interferons [42]. However, the differences between NK cells and NCCs should not be ignored, including the absence of homologs of NK cell markers in fish, such as CD56. Also, marked variation in the existence and functions of fish homologs of key molecules of NK cells. For instance, CD27 was not detected in tilapia NCCs [14]. Besides, our previous studies suggested that the lectins might play a pivotal role in the recognition of NCCs, while the CD94-mediated regulation is inconspicuous [19,20]. To summarize, tilapia NCCs most likely function as a distinct innate immune cell type.

In contrast, Mos/Mφs exhibited significant heterogeneity in response to the two bacterial species, with numerous branch-specific molecules being identified. Several classical proinflammatory cytokines, including TNF-α and IL-1β, likely played central roles in Mos/Mφs differentiation against Gram-negative bacteria. This is consistent with previous findings showing that *A. hydrophila* infection induced a stronger inflammatory response in tilapia head kidney [43]. However, second-messenger systems, such as AD and PKC, appear to play critical roles in mediating Mos/Mφs differentiation following Gram-positive bacterial infection. These pathways likely exert profound effects on cell fate and warrant urgent further investigation. In addition, given that these findings are based on a single post-infection time point and lack functional experimental validation of key discoveries, such as the distinct differentiation trajectories of Mos/Mφs. Future research should involve dynamic analyses at multiple time points and conduct functional experiments on the identified key genes to elucidate their specific immune mechanisms.

## 5. Conclusions

In summary, our findings confirm the central defensive roles of NCCs and Mos/Mφs, and uniquely reveal the distinct, pathogen-specific differentiation trajectories of Mos/Mφs in response to *S. agalactiae* and *A. hydrophila*. These insights provide a fundamental resource for understanding antibacterial immunity in fish, with direct implications for improving aquaculture health management and sustainability in China and globally. Furthermore, as *S. agalactiae* is a major human pathogen, our tilapia model offers valuable evolutionary perspectives on conserved immune pathways, contributing to the broader understanding of host–pathogen interactions and informing the development of novel antibacterial strategies for both animal and human health.

## Data Availability

The names of the repository/repositories and accession number(s) can be found below: https://www.ncbi.nlm.nih.gov/, PRJNA1304741 & PRJNA1304859 (accessed on 13 August 2025).

## Supplementary Materials

The following supporting information can be downloaded at: https://www.mdpi.com/article/10.3390/ani15202951/s1. Table S1: Differential differentiation genes highly correlated with NCC antibacterial response; Table S2: Differential differentiation genes around branch node 2 of Mo. Figure S1: UAMP of markers.

## References

[1] Preenanka R., Safeena M.P. (2023). Morphological, biological and genomic characterization of lytic phages against *Streptococcus agalactiae* causing streptococcosis in tilapia. Microb. Pathog..

[2] Zhang Z. (2021). Research advances on tilapia streptococcosis. Pathogens.

[3] AlYahya S.A., Ameen F., Al-Niaeem K.S., Al-Sa’adi B.A., Hadi S., Mostafa A.A. (2018). Histopathological studies of experimental *Aeromonas hydrophila* infection in blue tilapia, *Oreochromis aureus*. Saudi J. Biol. Sci..

[4] Liu G., Zhu J., Chen K., Gao T., Yao H., Liu Y., Zhang W., Lu C. (2016). Development of *Streptococcus agalactiae* vaccines for tilapia. Dis. Aquat. Organ..

[5] Pau E.J.J.N., Yong C.C. (2025). Input–output analysis of *Streptococcus* disease impact on Malaysian tilapia production and exports. Aquacult. Int..

[6] Raabe V.N., Shane A.L. (2019). Group B streptococcus (*Streptococcus agalactiae*). Microbiol. Spectr..

[7] Hou X., Li Q. (2024). Medulla oblongata and NCCs are central defenders against *Streptococcus agalactiae* infection of the tilapia brain. Front. Immunol..

[8] Hou X., Shi H., Jiang Y., Li X., Chen K., Li Q., Liu R. (2023). Transcriptome analysis reveals the neuroactive receptor genes response to *Streptococcus agalactiae* infection in tilapia, *Oreochromis niloticus*. Fish Shellfish Immunol..

[9] Li Q., Liu R., Ma R., Huang Y., Zhang Z., Zhang L., Zheng Z., Li X., Chen K., Chen C. (2022). Brain transcriptome response to *Streptococcus agalactiae* infection and the heterogeneous regulation of neuropeptides on immune response in tilapia, *Oreochromis niloticus*. Aquaculture.

[10] Fan B., Chen F., Li Y., Wang Z., Wang Z., Lu Y., Wu Z., Jian J., Wang B. (2019). A comprehensive profile of the tilapia (*Oreochromis niloticus*) circular RNA and circRNA–miRNA network in the pathogenesis of meningoencephalitis of teleosts. Mol. Omics.

[11] Zhang L., Wang C., Liu H., Fu P. (2019). The important role of phagocytosis and interleukins for Nile tilapia (*Oreochromis niloticus*) to defense infection of *Aeromonas hydrophila* based on transcriptome analysis. Fish Shellfish Immunol..

[12] Cui M., Wang Z., Yang Y., Liu R., Wu M., Li Y., Zhang Q., Xu D. (2022). Comparative transcriptomic analysis reveals the regulated expression profiles in *Oreochromis niloticus* in response to coinfection of *Streptococcus agalactiae* and *Streptococcus iniae*. Front. Genet..

[13] Wang B., Thompson K.D., Wangkahart E., Yamkasem J., Bondad-Reantaso M.G., Tattiyapong P., Jian J., Surachetpong W. (2023). Strategies to enhance tilapia immunity to improve their health in aquaculture. Rev. Aquac..

[14] Jiang B., Li Q., Zhang Z., Huang Y., Wu Y., Li X., Huang M., Huang Y., Jian J. (2023). Involvement of CD27 in innate and adaptive immunities of Nile tilapia (*Oreochromis niloticus*). Fish Shellfish Immunol..

[15] Mokhtar D.M., Zaccone G., Alesci A., Kuciel M., Hussein M.T., Sayed R.K. (2023). Main components of fish immunity: An overview of the fish immune system. Fishes.

[16] Lieschke G.J., Trede N.S. (2009). Fish immunology. Curr. Biol..

[17] Li Q., Jiang B., Zhang Z., Huang Y., Xu Z., Chen X., Hou X., Cai J., Huang Y., Jian J. (2022). Serotonin system is partially involved in immunomodulation of Nile tilapia (*Oreochromis niloticus*) immune cells. Front. Immunol..

[18] Wu L., Gao A., Li L., Chen J., Li J., Ye J. (2021). A single-cell transcriptome profiling of anterior kidney leukocytes from Nile tilapia (*Oreochromis niloticus*). Front. Immunol..

[19] Niu J., Huang Y., Liu X., Zhang Z., Tang J., Wang B., Lu Y., Cai J., Jian J. (2020). Single-cell RNA-seq reveals different subsets of non-specific cytotoxic cells in teleost. Genomics.

[20] Li Q., Jiang B., Zhang Z., Huang Y., Xu Z., Chen X., Huang Y., Jian J., Yan Q. (2022). Involvement and characterization of NLRCs and pyroptosis-related genes in Nile tilapia (*Oreochromis niloticus*) immune response. Fish Shellfish Immunol..

[21] Li Q., Jiang B., Zhang Z., Huang Y., Xu Z., Chen X., Huang Y., Jian J., Yan Q. (2022). α-MSH is partially involved in the immunomodulation of Nile tilapia (*Oreochromis niloticus*) antibacterial immunity. Fish Shellfish Immunol..

[22] Zhang Z., Li X., Huang M., Huang Y., Tan X., Dong Y., Huang Y., Jian J. (2024). Siglec7 functions as an inhibitory receptor of non-specific cytotoxic cells and can regulate the innate immune responses in a primitive vertebrate (*Oreochromis niloticus*). Int. J. Biol. Macromol..

[23] Zhang Z., Niu J., Li Q., Huang Y., Jiang B., Li X., Jian J., Huang Y. (2022). A novel C-type lectin (CLEC12B) from Nile tilapia (*Oreochromis niloticus*) is involved in host defense against bacterial infection. Fish Shellfish Immunol..

[24] Duan H., Zhang Y., Liu J., Ren G., Li Z., Tian Y. (2025). Transcriptome analysis reveals the DNA replication genes response to *Vibrio anguillarum* and NNV infection in Jinhu grouper (*Epinephelus fuscoguttatus*♀× *Epinephelus tukulal*♂). Comp. Biochem. Physiol. Part. D Genomics Proteomics.

[25] Ken C.-F., Chen C.-N., Ting C.-H., Pan C.-Y., Chen J.-Y. (2017). Transcriptome analysis of hybrid tilapia (*Oreochromis* spp.) with *Streptococcus agalactiae* infection identifies Toll-like receptor pathway-mediated induction of NADPH oxidase complex and piscidins as primary immune-related responses. Fish Shellfish Immunol..

[26] Jia R., Hou Y., Zhou L., Zhang C., Li B., Zhu J. (2025). Combined effects of high-fat diet feeding and *Streptococcus agalactiae* infection on lipid metabolism, antioxidant status, and immune response in tilapia (*Oreochromis niloticus*). Comp. Biochem. Physiol. C Toxicol. Pharmacol..

[27] Guo S., Gao W., Zeng M., Liu F., Yang Q., Chen L., Wang Z., Jin Y., Xiang P., Chen H. (2023). Characterization of TLR1 and expression profiling of TLR signaling pathway related genes in response to *Aeromonas hydrophila* challenge in hybrid yellow catfish (*Pelteobagrus fulvidraco*♀× *P*. vachelli♂). Front. Immunol..

[28] Zhan F.-B., Jakovlić I., Wang W.-M. (2019). Identification, characterization and expression in response to *Aeromonas hydrophila* challenge of five interferon regulatory factors in *Megalobrama amblycephala*. Fish Shellfish Immunol..

[29] Wang X., Wu Z., Wu S., Chen X., Hanif M., Zhang S. (2021). Hematological and cytochemical characteristics of peripheral blood cells in the argus snakehead (*Ophiocephalus argus Cantor*). PeerJ.

[30] Megarani D.V., Hardian A.B., Arifianto D., Santosa C.M., Salasia S.I. (2020). Comparative morphology and morphometry of blood cells in zebrafish (*Danio rerio*), common carp (*Cyprinus carpio carpio*), and tilapia (*Oreochromis niloticus*). J. Am. Assoc. Lab. Anim. Sci..

[31] Bai H., Mu L., Qiu L., Chen N., Li J., Zeng Q., Yin X., Ye J. (2022). Complement C3 regulates inflammatory response and Monocyte/Macrophage phagocytosis of *Streptococcus agalactiae* in a teleost fish. Int. J. Mol. Sci..

[32] Huang Y., Chen Z., Xie R., Wang P., Zhang Z., Cai J., Wang B., Jian J. (2022). Transferrin mediated NCC killing activity through NCCRP-1 in Nile tilapia (*Oreochromis niloticus*). Fishes.

[33] Monir M.S., Yusoff M.S.M., Zamri-Saad M., Amal M.N.A., Mohamad A., Azzam-Sayuti M., Ina-Salwany M.Y. (2022). Effect of an oral bivalent vaccine on immune response and immune gene profiling in vaccinated red tilapia (*Oreochromis* spp.) during infections with *Streptococcus iniae* and *Aeromonas hydrophila*. Biology.

[34] Guo X., Dang H., Huang W., Hassan Z., Yun S., Lu Y., Wang J., Zou J. (2024). IL-20 is produced by CD3γδ T cells and induced in the mucosal tissues of grass carp during infection with *Aeromonas hydrophila*. Dev. Comp. Immunol..

[35] Tian T., Wang J., Pan Y., Han X., Hu Y., Li J., Zhang Y., Zhang X. (2025). Chinese yam polysaccharide induces the differentiation and natural antibody secretion of IgM+ B cells to prevent *Aeromonas hydrophila* infection in grass carp. Int. J. Biol. Macromol..

[36] Shimon-Hophy M., Jacob A., Avtalion R.R. (2020). NCCRP-1 might not be a marker of so called NCC cells in common carp (*Cyprinus carpio*) leukocytes. Am. J. Immunol..

[37] Kallio H., Tolvanen M., Jänis J., Pan P.-w., Laurila E., Kallioniemi A., Kilpinen S., Tuominen V.J., Isola J., Valjakka J. (2011). Characterization of non-specific cytotoxic cell receptor protein 1: A new member of the lectin-type subfamily of F-box proteins. PLoS ONE.

[38] Yao Y., Li Q., Yan Q. (2024). Distribution and response strategies of non-specific cytotoxic cell receptor protein 1 in large yellow croaker. Fish Shellfish Immunol..

[39] Zhang X., Zhao H., Chen Y., Luo H., Yang P., Yao B. (2015). A zebrafish (*Danio rerio*) bloodthirsty member 20 with E3 ubiquitin ligase activity involved in immune response against bacterial infection. Biochem. Biophys. Res. Commun..

[40] Jang J.H., Jung I.Y., Kim H., Cho J.H. (2022). Rainbow trout USP4 downregulates LPS-induced inflammation by removing the K63-linked ubiquitin chain on TAK1. Fish Shellfish Immunol..

[41] Graves S.S., Evans D.L., Cobb D., Dawe D.L. (1984). Nonspecific cytotoxic cells in fish (*Ictalurus punctatus*) I. Optimum requirements for target cell lysis. Dev. Comp. Immunol..

[42] Mali P., Sanyal K.B., Mukherjee D., Guchhait A., Dash G. (2017). Nonspecific cytotoxic cells (NCC) in fish: A review. J. Interacad..

[43] Li Q., Jiang B., Zhang Z., Huang Y., Xu Z., Chen X., Cai J., Huang Y., Jian J. (2022). CRP Involved in Nile tilapia (*Oreochromis niloticus*) against bacterial infection. Biology.

