# Supplementary figures and images for: RNA-Seq and Single-Cell RNA-Seq Analyses of Tilapia Head Kidney in Response to Streptococcus agalactiae and Aeromonas hydrophila
Source: Animals (Basel). 2025 Oct 11;15(20):2951. doi: 10.3390/ani15202951 (PMC12560893; doi:10.3390/ani15202951)

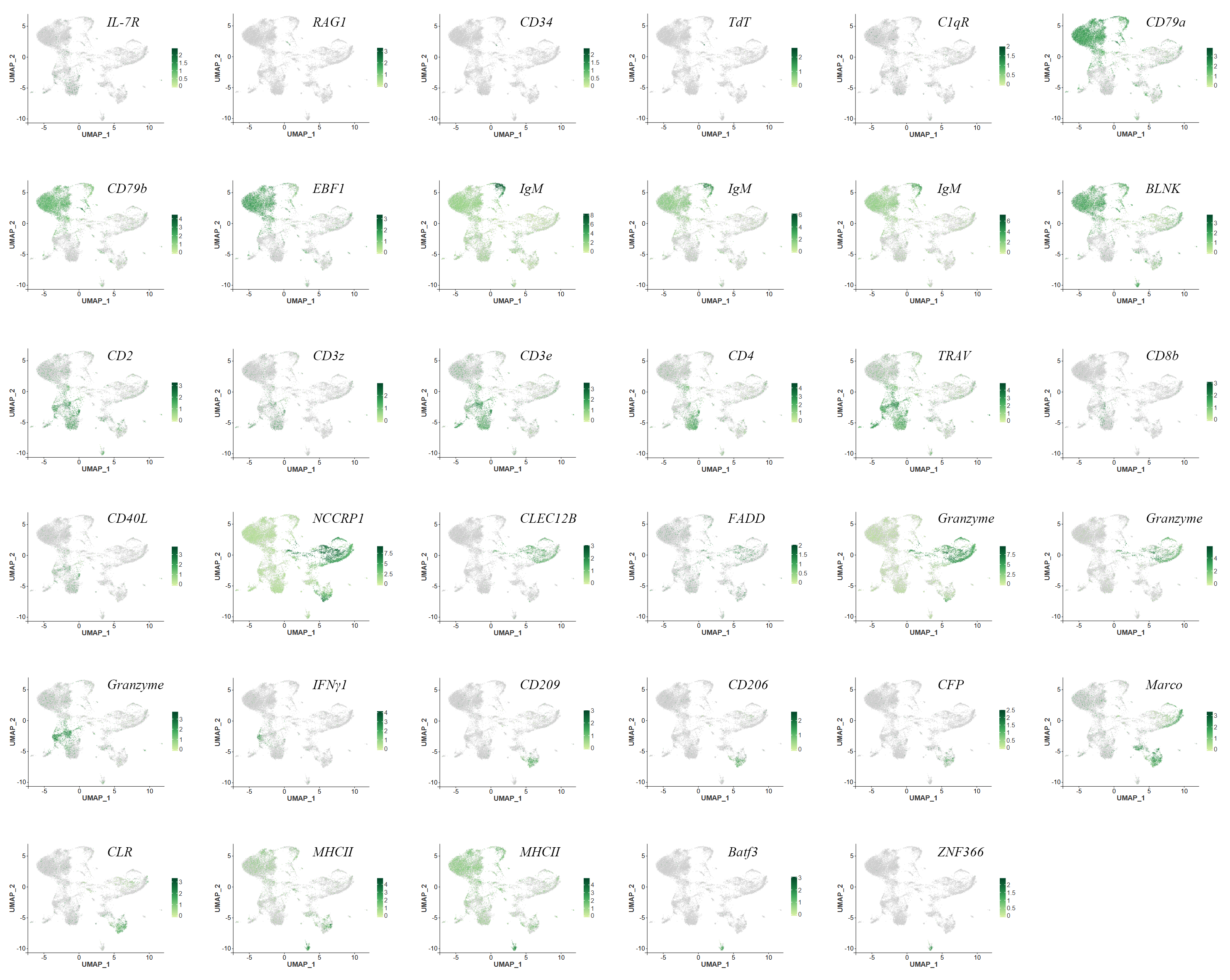

Supplement: Supplementary file 1 [file animals-15-02951-s001.zip › Figure S1. UAMP of markers.tif]
